# Apatinib combined with PD-1 antibody for third-line or later treatment of advanced gastric cancer

**DOI:** 10.3389/fonc.2022.952494

**Published:** 2022-10-28

**Authors:** Qingli Cui, Yuefeng Mao, Daoyuan Wu, Yanhui Hu, Dongyang Ma, LiHan Zhang, Huaimin Liu

**Affiliations:** ^1^ Department of Integrated Traditional Chinese and Western Medicine, Affiliated Cancer Hospital of Zhengzhou University & Henan Cancer Hospital, Zhengzhou, Henan, China; ^2^ Department of Medical Oncology, Second People’s Hospital of Pingdingshan, Pingdingshan, Henan, China; ^3^ Department of Pathology, Affiliated Cancer Hospital of Zhengzhou University & Henan Cancer Hospital, Zhengzhou, Henan, China

**Keywords:** gastric cancer, prognosis, apatinib, PD-1 mAb, treatment, antiangiogenesis, immunotherapy

## Abstract

**Background:**

Both apatinib and programmed death 1 (PD-1) monoclonal antibody (mAb) monotherapy have been licensed in China for the third-line treatment of advanced gastric cancer (AGC). However, whether the combination could improve the prognosis of patients with AGC after second-line treatment has not been evaluated.

**Methods:**

We retrospectively screened 892 patients with AGC who received third-line or later treatment from June 2016 to July 2021 at the Affiliated Cancer Hospital of Zhengzhou University and second People’s Hospital of Pingdingshan. 166 patients who received apatinib plus PD-1 mAb, apatinib, or PD-1 mAb were included. Based on medical records and follow-up data, we analyzed the efficacy and safety of these three treatment options.

**Results:**

Patients received apatinib plus PD-1 mAb (n=49), apatinib monotherapy (n=63), or PD-1 mAb monotherapy (n=54). Apatinib plus PD-1 mAb showed significantly longer progression-free survival (PFS) and overall surivival (OS) compared with the apatinib monotherapy (PFS: 5.5 months versus 3.0 months; *p*=0.002; OS: 10 months versus 7.6 months; *p*=0.011) or PD-1 mAb monotherapy (PFS: 5.5 months versus 2.3 months; *p*=0.017; OS: 10 months versus 6.5 months; *p*=0.004). Apatinib plus PD-1 mAb showed higher ORR and DCR than the apatinib and PD-1 mAb monotherapy (ORR: 34.7% versus 6.3% versus 9.3%; p=0.001; DCR: 75.5% versus 44.4% versus 40.7%; p=0.001). Further subgroup analysis for PFS and OS shown consistent efficacy in most subgroups with apatinib plus PD-1 mAb versus apatinib monotherapy or PD-1 mAb monotherapy. Multivariate analyses suggested that apatinib plus PD-1 mAb was significantly associated with better PFS and OS. Most of the treatment-related toxicities were mild and tolerable.

**Conclusion:**

Compared with the monotherapy of either apatinib or PD-1 mAb, apatinib plus PD-1 mAb treatment yielded longer PFS and OS, and achieved significant higher ORR and DCR.

## Introduction

Gastric cancer (GC) remains an important cancer worldwide, ranking fifth for incidence and fourth for mortality (1). Due to late diagnosis and poor therapeutic efficacy, mortality from gastric cancer is high (2). Although standard treatment including cytotoxic agents and molecular targeted therapies has improved survival in patients with advanced gastric cancer (AGC) by around 1 year, the prognosis remains poor (3–7). The backbone of therapy against AGC in first line remains platinum-based chemotherapy combination regimens (with trastuzumab for HER-2-positive patients; with nivolumab for HER-2-negetive patients) (3, 4, 6, 8). In the second line, ramucirumab, a vascular endothelial growth factor receptor (VEGFR) inhibitor, can be administered as a single agent or in combination with taxane (5, 7), and the programmed death 1 (PD-1) monoclonal antibody (mAb) pembrolizumab is approved for patients with microsatellite instability-high (MSH) tumours (9). As for the third-line or later treatment, both apatinib and PD-1 mAb have been approved in China (10–12). However, although apatinib was demonstrated with benefits in both progression-free survival (PFS) and overall survival (OS) compared with placebo, the objective response rate (ORR) was only 2.8% and the PFS was only 2.6 months (10). In the ATTRACTION-2 study and KENOTE-059 study, the ORR with PD-1 mAb were limited to around 11% and PFS were limited to around 2 months (11, 12). Therefore, there remained an unmet need for effective therapies for AGC.

The combination of targeted therapy with immunotherapy is a new research field in the treatment of cancers. Apatinib is an oral multi-target drug which could block VEGFR-2 and inhibit tumor growth and metastasis (10, 13). Interestingly, one study has shown combined apatinib and PD-L1 blockade therapy synergistically enhances antitumor immune responses and promotes high endothelial venules formation in GC (14). Another study has shown PD-1 inhibitor combined with apatinib could modulate the tumor microenvironment and potentiate anti-tumor effect in mice bearing GC (15). Additionally, in a phase I study of PD-1 mAb (SHR-1210) combined with apatinib for advanced hepatocellular carcinoma, gastric or esophagogastric junction cancer, 25 of 43 patients with AGC achieved an ORR of 17.4% and DCR of 78.3%. In this study, PD-1 mAb (SHR-1210) plus apatinib achieved a median PFS of 2.9 months and a median OS of 11.4 months (16). In a phase 2 study, PD-1 mAb (SHR-1210) combined with apatinib and S-1 as second-line treatment for AGC achieved an ORR of 29.2% and a median PFS of 6.5 months (17). However, the combination of apatinib and PD-1 mAb has not been investigated in third-line or later treatment of patients with AGC. Therefore, we performed a multicenter retrospective analysis to compare the efficacy between apatinib plus PD-1 mAb and apatinib or PD-1 mAb monotherapy in patients with AGC as third-line or later treatment.

## Materials and methods

### Patients

In this retrospective study, patients with AGC who received apatinib plus PD-1 mAb and either apatinib or PD-1 mAb monotherapy as the third-line or later treatment between June 2016 and July 2021 were identified from the Affiliated Cancer Hospital of Zhengzhou University and Second People’s Hospital of Pingdingshan. Patient characteristics and survival outcomes were retrospectively retrieved from medical records and follow-up data. The eligibility criteria were as follows: pathologically confirmed AGC; no prior treatment with apatinib or PD-1 mAb; adequate organ function; Eastern Cooperative Oncology Group performance status (ECOG PS) of 0–2; no bleeding disorders; with at least a measurable lesion; and with complete efficacy evaluation or follow-up data. Major exclusion criteria included history of previous treatment with apatinib, PD-1 mAb, or anti-PD-L1 monoclonal antibodies; autoimmune disease; or the presence of a serious comorbidity, such as intestinal obstruction, pulmonary fibrosis, and inadequate organ function.

This retrospective study was approved by the ethics committee of Affiliated Cancer Hospital of Zhengzhou University and second People’s Hospital of Pingdingshan and was implemented in accordance with the guidelines in Declaration of Helsinki. Because this was a retrospective study, informed consent was waived.

### Molecular characteristics

Molecular characteristics such as human epidermal growth factor receptor-2 (HER-2), mismatch repair (MMR) and programmed cell death ligand 1 (PD-L1) were obtained by reviewing the results of immunohistochemistry and molecular pathology. HER-2 status was performed by immunohistochemistry (IHC) or fluorescence *in situ* hybridization (FISH). HER-2 positivity was defined as an IHC score of 3+ or an IHC score of 2+ and a FISH positive. PD-L1 expression was measured by combined positive score (CPS) and assessed by IHC using an anti-PD-L1 rabbit monoclonal antibody (Clone 22C3 or SP263). The CPS score was defined as the proportion of PD-L1-positive cells (tumor cells, lymphocytes and macrophages) multiplied by 100. MMR system (MLH1, MSH2, MSH6 and PMS2) was assessed by IHC, and tumors that lacked either MLH1, MSH2, MSH6 or PMS2 expression were considered as MMR-deficient, and those maintained expression of MLH1, MSH2, MSH6 and PMS2 were considered as MMR-proficient.

### Treatment

In this study, PD-1 mAb included nivolumab, pembrolizumab, camrelizumab, sintilimab and toripalimab. PD-1 mAbs were administered at the following dose: nivolumab 240mg intravenous every 2 weeks or 360mg intravenous every 3 weeks, pembrolizumab, camrelizumab, sintilimab and toripalimab 200mg intravenous every 3 weeks. Patients in apatinib plus PD-1 mAb group received 250 mg oral apatinib daily and intravenous PD-1 mAb every 2 or 3 weeks. Patients in apatinib monotherapy group received 500 mg oral apatinib daily for 4 weeks as a cycle. Patients in PD-1 mAb group received intravenous PD-1 mAb every 2 or 3 weeks. Treatment was continued until patients experienced intolerable toxicity or disease progression. Dose modification was allowed for drug-related toxicity.

### Statistical analysis

In this study, all outcomes were compared between apatinib plus PD-1 mAb and apatinib or PD-1 mAb monotherapy. Patient baseline characteristics and response rates were compared using χ^2^ test or Fisher’s exact test. Treatment responses, including partial response (PR), complete response (CR), stable disease (SD) and progressive disease (PD), were assessed according to the Response Evaluation Criteria in Solid Tumors (RECIST) version 1.1 every 2 or 3 cycles. The objective response rate (ORR) was defined as the proportion of CR plus PR. The disease control rate (DCR) was defined as the proportion of CR, PR, and SD. PFS was calculated from the beginning of study treatment to disease progression or death. OS was calculated from the initiation of study treatment to death from any cause. We assessed PFS and OS by the Kaplan-Meier method, compared between groups by the log-rank test. Hazards ratios (HRs) were determined using the Cox proportional hazards models.

Patient baseline characteristics were used as covariates in the analysis, including sex, age, tumor primary site, histology, ECOG PS, previous gastrectomy, previous treatment, metastatic site, HER-2 status and PD-L1 CPS expression. Subgroup analyses for PFS and OS were performed according to the baseline factors. Univariate analysis and multivariate Cox regression model were used to identify the impact factors of PFS and OS. Variables with *p*-value thresholds <0.20 determined by univariate analysis were entered into a Cox proportional hazards model for multivariate analysis. Adverse events were evaluated according to National Cancer Institute Common Terminology Criteria for Adverse Events (NCI CTCAE) version 4.0. All statistical analyses were performed using SPSS version 23.0 (IBM, New York, USA) and Graphpad prism (version 8.0.1 GraphPad Software Inc., San Diego, CA, USA). All *p*-value less than 0.05 was considered statistically significant.

## Results

### Patient characteristics

**Figure 1:**
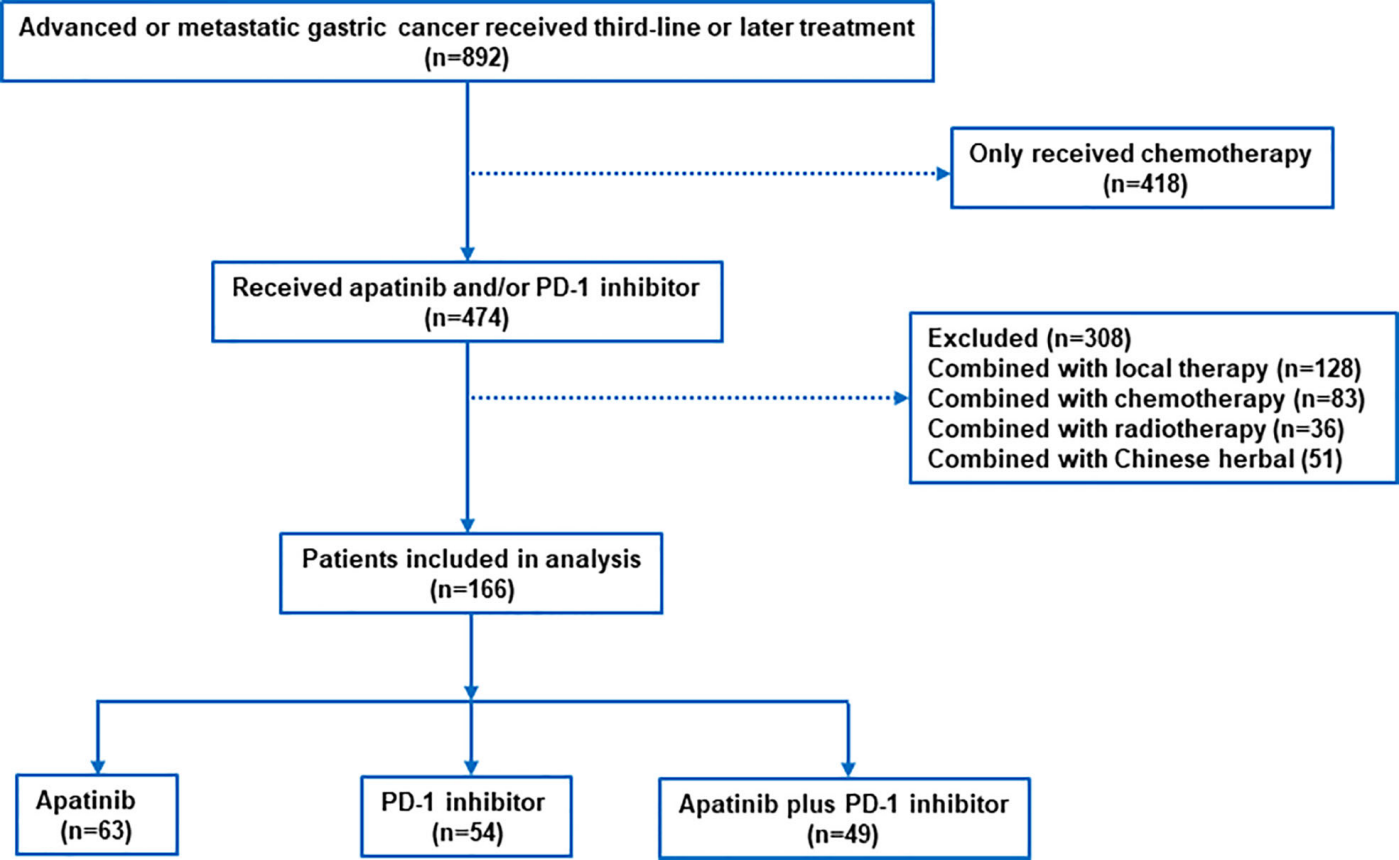
Patient inclusion flowchart.

Between June 2016 and July 2021, 892 patients with advanced AGC were screened at the study centers, of which 166 patients met the inclusion criteria (apatinib plus PD-1 mAb, n=49; apatinib, n=63; PD-1 mAb n=54; **Figure 1**). The median age was 64 years (range, 26-79) and 112 (67.5%) patients were male. The most common histology type was diffuse (n=107, 64.5%), and the most common location of primary tumor was the gastric (n=110, 66.3%). Majority of patients (n=126, 75.9%) had ECOG PS of 0 or 1. Only 67 (40.4%) patients had received gastrectomy. Overall, 155 (93.4%) patients had received two previous lines of treatment, and 6.6% had received at least three lines of treatment. The most common metastatic sites were the lymph nodes, liver, peritoneum and lungs. All patients had measurable lesions. Baseline clinical characteristics were exhibited in **Table 1**. Data on HER-2, MMR status and PD-L1 CPS were available in 162, 151 and 119 patients. There was no significant difference between the three groups, except for MMR status and PD-L1 CPS expression.

**Table 1:** Patient baseline characteristics.

Variables	Apatinib plus PD-1 mAb (n=49)	Apatinib (n=63)	PD-1 mAb (n=54)	*p* value
**Age**				
** Median (rang)**	63 (26-78)	64 (30-79)	64 (35-79)	
≥65 years, n (%)	19 (38.8)	25 (39.7)	22 (40.7)	0.979
<65 years, n (%)	30 (61.2)	38 (60.3)	32 (59.3)	
**Gender, n (%)**				
Male	32 (65.3)	42 (66.7)	38 (70.4)	0.848
Female	17 (34.7)	21 (33.3)	16 (29.6)	
**Primary site, n (%)**				
Gastric	34 (69.4)	40 (63.5)	36 (66.7)	0.805
Gastroesophageal	15 (30.6)	23 (36.5)	18 (33.3)	
**Histology, n (%)**				
Diffuse	30 (61.2)	44 (69.8)	33 (61.1)	0.526
Intestinal	19 (38.3)	19 (30.2)	21 (38.9)	
**ECOG PS, n (%)**				
0-1	35 (71.4)	45 (71.4)	46 (85.2)	0152
2	14 (28.6)	18 (28.6)	8 (14.8)	
**Previous gastrectomy, n (%)**	15 (30.6)	28 (44.4)	24 (44.4)	0.253
**Previous lines of treatment, n (%)**				
2	45 (91.8)	61 (96.8)	49 (90.7)	0.367
>2	4 (8.2)	2 (3.2)	5 (9.3)	
**Site of metastasis, n (%)**				
Lymph node	38 (77.6)	49 (77.8)	39 (72.2)	0.743
Liver	21 (42.9)	30 (47.6)	20 (37)	0.514
Peritoneum	15 (30.6)	19 (30.2)	20 (37)	0.690
Lung	7 (14.3)	12 (19)	10 (18.5)	0.781
Other	17 (34.7)	16 (25.4)	14 (25.9)	0.497
**Her-2 status, n (%)**				
Positive	9 (18.4)	10 (15.9)	12 (22.2)	0.124
Negetive	40 (81.6)	49 (77.8)	42 (77.8)	
Unknown	0 (0.0)	4 (6.3)	0 (0.0)	
**MMR status, n (%)**				
MMR-proficient	49 (100)	48 (76.2)	53 (98.1)	0.001
MMR-deficient	0 (0.0)	0 (0.0)	1 (1.9)	
Unknown	0 (0.0)	15 (23.8)	0 (0.0)	
**PD-L1 CPS, n (%)**				
<1	28 (57.1)	13 (20.6)	32 (59.3)	0.001
≥1	20 (40.8)	7 (11.1)	19 (35.2)	
Unknown	1 (2)	43 (68.3)	3 (5.6)	

ECOG PS, eastern cooperative oncology group performance status; Her-2, human epidermal growth factor receptor 2; MMR, mismatch repair; CPS, combined positive score.

### Efficacy

**Figure 2:**
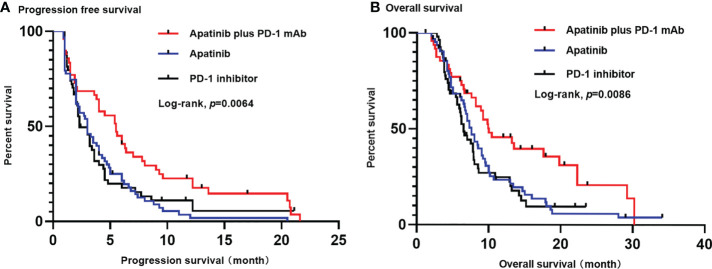
Kaplan-Meier estimates of PFS **(A)** and OS **(B)** for each group. **(A)** The median PFS was significantly longer in apatinib plus PD-1 mAb than in apatinib monotherapy (5.5 months versus 3.0 months; p=0.002) or in PD-1 mAb monotherapy (5.5 months versus 2.3 months; p=0.017). **(B)** The OS was significantly longer in apatinib plus PD-1 mAb than in apatinib monotherapy (10 months versus 7.6 months; p=0.011) or in PD-1 mAb group (10 months versus 6.5 months; p=0.004). PFS, progression-free survival. OS, progression-free survival; CI: confidence interval.

At the time of the analysis (1 January 2022), the median follow-up time was 24.7 months (rang 2.3-42.9 months). The median PFS and OS in the overall population were 3.3 months (95% CI, 2.7-3.9 months) and 7.8 months (95% CI, 6.8-8.8 months), respectively. The apatinib plus PD-1 mAb group showed significantly longer PFS [median 5.5 months (95% CI, 3.7-7.3 months) *versus* 3.0 months (95% CI, 2.3-3.7 months); HR=0.56 (95% CI, 0.38-0.82); *p*=0.002] and OS [median 10 months (95% CI, 5.3-13.7 months) *versus* 7.6 months (95% CI, 6.5-8.7 months); HR=0.59 (95% CI, 0.39-0.89); *p*=0.011] compared with the apatinib monotherapy group. Likewise, apatinib plus PD-1 mAb showed significantly longer PFS [median 5.5 months (95% CI, 3.7-7.3 months) *versus* 2.3 months (95% CI, 1.4-3.2 months); HR=0.62 (95% CI, 0.41-0.95); *p*=0.017] and OS [median 10 months (95% CI, 5.3-14.7 months) *versus* 6.5 months (95% CI, 5.5-7.5 months); HR=0.54 (95% CI, 0.34-0.84); *p*=0.004] compared with the PD-1 mAb monotherapy group (**Figure 2**). In addition, the apatinib plus PD-1 mAb group showed higher ORR and DCR than the apatinib monotherapy (ORR: 34.7% *versus* 6.3%; *p*<0.001; DCR: 75.5% *versus* 44.4%; *p*=0.001) and PD-1 mAb monotherapy (ORR: 34.7% *versus* 9.3%; *p*=0.002; DCR: 75.5% versus 40.7%; p<0.001). Patient’s response to treatment was displayed in the **Table 2**.

**Table 2:** Overall response.

Best response n (%)	Apatinib plus PD-1 mAb (n = 49)	Apatinib (n = 63)	PD-1 mAb (n = 54)	*p* value
PR	17 (34.7)	4 (6.3)	5 (9.3)	
SD	20 (40.8)	24 (38.1)	17 (31.5)	
PD	12 (24.5)	35 (55.6)	32 (59.3)	
ORR	17 (34.7)	4 (6.3)	5 (9.3)	<0.001
DCR	37 (75.5)	28 (44.4)	22 (40.7)	0.001

PR, partial response; SD, stable disease; PD, progressive disease; ORR, overall response rate; DCR, disease control rate.

**Figure 3:**
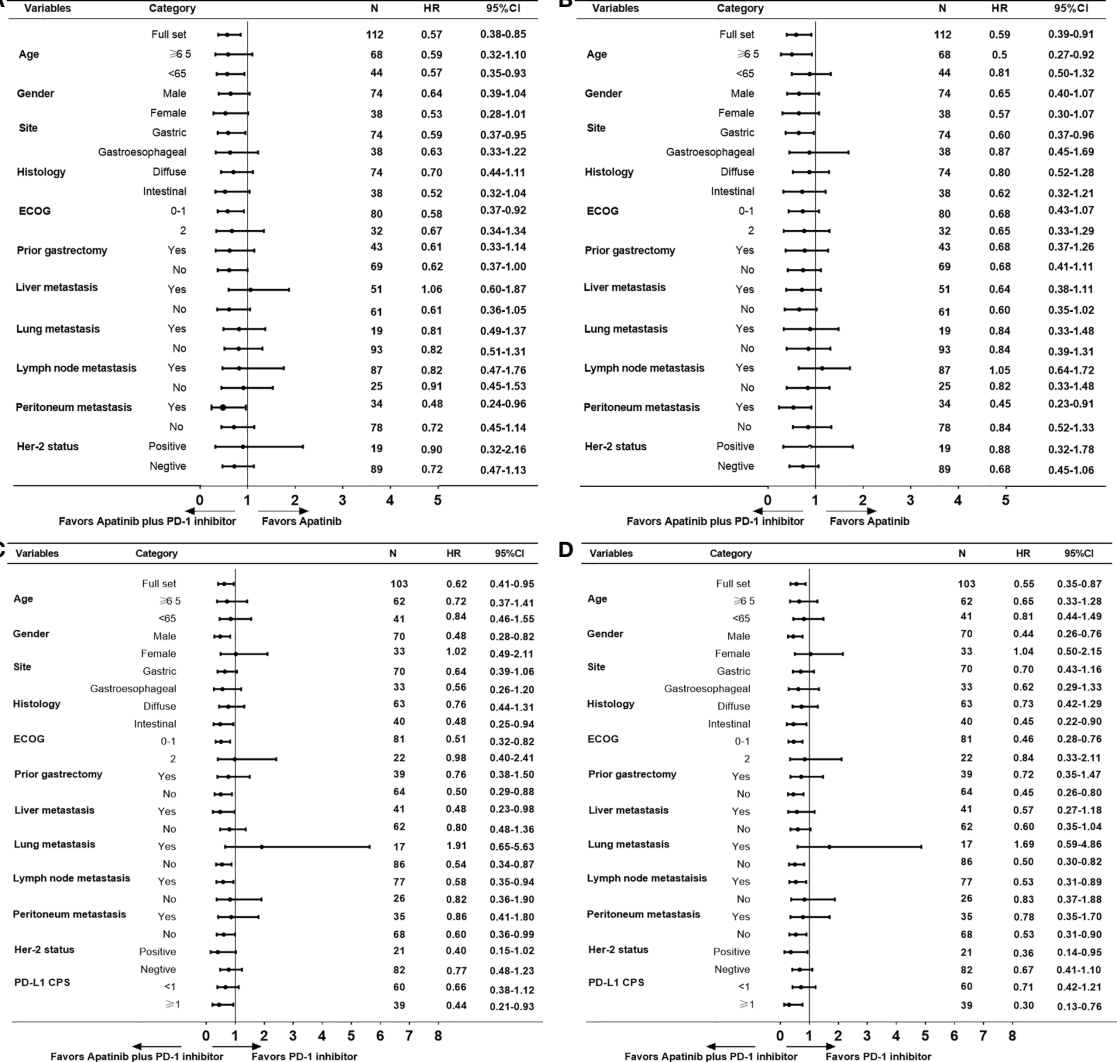
Subgroup analyses for PFS and OS. **(A)** PFS and **(B)** OS for apatinib plus PD-1 mAb *versus* apatinib monotherapy. **(C)** PFS and **(D)** OS for apatinib plus PD-1 mAb *versus* PD-1 mAb. PFS, progression-free survival. OS, progression-free survival.

Further subgroup analysis for PFS and OS shown that apatinib plus PD-1 mAb was more effective than apatinib monotherapy or PD-1 mAb monotherapy in most subgroups (**Figure 3**). Further breakdown of the subgroup of patients with metastasis by the site of metastasis, apatinib plus PD-1 mAb showed no PFS benefit for patients who had liver metastasis (HR=1.06, 95% CI, 0.60-1.87; **Figure 3A**] and no OS benefit for patients who had lymph nodes metastasis (HR=1.05, 95% CI, 0.64-1.72; **Figure 3B**) than the apatinib monotherapy. In female patients, apatinib plus PD-1 mAb showed no advantage in PFS and OS compared with PD-1 mAb monotherapy (PFS : HR=1.02, 95% CI, 0.49-2.11; OS: HR=1.04, 95% CI, 0.50-2.15; **Figures 3C, D**). In patients with lung metastasis, apatinib plus PD-1 mAb showed a shorter PFS and OS than the PD-1 mAb monotherapy (PFS : HR=1.91, 95% CI, 0.65-5.63; OS: HR=1.69, 95% CI, 0.59-4.86; **Figures 3C, D** ). However, there were only 17 patients with lung metastasis received apatinib plus PD-1 mAb or PD-1 mAb monotherapy, caution should be taken when drawing conclusions.

To further identify the impact factors of PFS and OS, firstly we performed a univariate analysis, and variables with *p*-value <0.20 were included in the multivariate analysis. The results of univariate analysis for PFS and OS are shown in **Supplement Table S1** (apatinib plus PD-1 mAb versus apatinib monotherapy) and S2 (apatinib plus PD-1 mAb versus PD-1 mAb monotherapy). Multivariate analyses results were listed in **Table 3** and **Table 4**. The results suggested that apatinib plus PD-1 mAb treatment was significantly associated with longer PFS and OS compared with apatinib monotherapy (PFS: HR=0.48, 95% CI, 0.32-0.74; *p*=0.001; OS: HR=0.48, 95% CI, 0.31-0.76; *p*=0.002) or PD-1 mAb monotherapy (PFS: HR=0.55, 95% CI, 0.34-0.87; *p*=0.011; OS: HR=0.46, 95% CI, 0.28-0.75; *p*=0.002). On multivariate analyses for apatinib plus PD-1 mAb versus apatinib monotherapy, ECOG PS of 0 or 1 was considered as an independent predictor of longer PFS and OS (PFS: HR=0.54, 95% CI, 0.35-0.83; p=0.005; OS: HR=0.48, 95% CI, 0.26-0.71; p=0.001), while presence of liver metastasis has detrimental effect on PFS and OS (PFS: HR=1.69, 95% CI, 1.31-2.52; p=0.005; OS: HR=1.72, 95% CI, 1.11-2.67; p=0.016). On multivariate analyses for apatinib plus PD-1 mAb versus PD-1 mAb monotherapy, PD-L1 CPS≥1 was considered as an independent protective prognosis factor of PFS and OS (PFS: HR=0.34, 95% CI, 0.21-0.54; *p*=0.001; OS: HR=0.22, 95% CI, 0.12-0.39; *p*=0.001), while ECOG PS of 0 or 1 has positive effect on OS (HR=0.48, 95% CI, 0.27-0.84; p=0.011).

**Table 3:** Multivariate analyses of progression-free survival and overall survival for apatinib plus PD-1 mAb *versus* apatinib monotherapy.

	Progression-free survival			Overall survival		
Variable	HR	95%CI	*p* value	HR	95%CI	*p* value
**Gender**						
Male	0.894	0.595-1.343	0.589	0.646	0.413-1.009	0.055
Female	Ref			Ref		
**Histology**						
Diffuse	—	—	—	1.078	0.675-1.720	0.754
Intestinal	—	—	—	Ref		
**ECOG**						
0-1	0.536	0.346-0.833	0.005	0.429	0.261-0.705	0.001
2	Ref			Ref		
**Site of metastasis**						
**Liver**	1.688	1.131-2.519	0.010	1.721	1.107-2.673	0.016
No	Ref			Ref		
**Peritoneum**	1.090	0.714-1.663	0.690	—	—	—
No	Ref			—	—	—
**Treatment**						
Apatinib plus PD-1 mAb	0.483	0.316-0.740	0.001	0.483	0.307-0.762	0.002
Apatinib	Ref			Ref		

HR, hazard ratio; CI, confidence interval; ECOG PS, eastern cooperative oncology group performance status; Ref, reference.

**Table 4:** Multivariate analyses of progression-free survival and overall survival for apatinib plus PD-1 mAb *versus* PD-1 mAb monotherapy.

	Progression-free survival			Overall survival		
Variable	HR	95%CI	*p* value	HR	95%CI	*p* value
**Gender**						
Male	—	—	—	1.075	0.641-1.802	0.784
Female	—	—	—	Ref		
**ECOG**						
0-1	0.597	0.346-1.030	0.064	0.477	0.271-0.841	0.011
2	Ref			Ref		
**Previous gastrectomy**						
Yes	—	—	—	0.628	0.377-1.046	0.074
No	—	—	—	Ref		
**Previous lines of treatment** chemotherapy, n (%)						
2	—	—	—	1.124	0.538-2.347	0.756
>2	—	—	—	Ref		
**Site of metastasis**						
**Liver**	1.036	0.644-1.669	0.883	—	—	—
No	Ref			—	—	—
**Lung**	1.613	0.925-2.812	0.092	1.962	0.750-2.698	0.055
No	Ref			Ref		
**PD-L1 CPS**						
≥1	0.338	0.213-0.537	0.001	0.221	0.124-0.394	0.001
<1	Ref			Ref		
**Treatment**						
Apatinib plus PD-1 mAb	0.545	0.341-0.869	0.011	0.461	0.284-0.746	0.002
PD-1 mAb	Ref			Ref		

HR, hazard ratio; CI, confidence interval; ECOG PS, eastern cooperative oncology group performance status; Ref, reference; CPS, combined positive score.

### Safety

The adverse events during the treatment were listed in the **Table 5**. Non-hematologic adverse events were more common than hematologic adverse events. Most of the treatment-related toxicities were mild and tolerable. No significant difference was observed in adverse events of any grade between apatinib plus PD-1 mAb and apatinib monotherapy groups (65.3% *versus* 68.3%; *p*=0.731). On the other hand, compared with the PD-1 mAb monotherapy group, the apatinib plus PD-1 mAb group showed more adverse events (65.3% *versus* 38.9%; *p*<0.001). The most common adverse events in apatinib plus PD-1 mAb group and apatinib monotherapy group of any grade were hypertentsion (40.8% veusus 42.9%), hand-foot syndrome (32.7% veusus 30.2%) and proteinuria (32.7% veusus 27%). The incidence of grade ≥3 adverse events was similar between apatinib plus PD-1 mAb and apatinib (34.7% *versus* 30.2%; *p*=0.612). The incidence of grade ≥3 adverse events with apatinib plus PD-1 mAb was significantly higher than that with PD-1 mAb monotherapy (34.7% *versus* 7.4%; *p*<0.001). No treatment related deaths were recorded.

**Table 5:** Treatment-related toxicities (n [%]).

	Apatinib plus PD-1 mAb (n = 49)		Apatinib (n = 63)		PD-1 mAb (n = 54)	
TRAEs	Any grade	Grade≥3	Any grade	Grade ≥3	Any grade	Grade≥3
Any events	32 (65.3)	17 (34.7)	43 (68.3)	19 (30.2)	21 (38.9)	4 (7.4)
Hypertension	20 (40.8)	6 (12.2)	27 (42.9)	9 (14.3)	1 (1.9)	0
Fatigue	14 (28.6)	2 (4.1)	16 (25.4)	1 (1.6)	5 (9.3)	0
Anorexia	13 (26.5)	1 (2.0)	15 (23.8)	0	6 (11.1)	1 (1.9)
Hand-foot syndrome	16 (32.7)	3 (6.1)	19 (30.2)	4 (6.3)	0	0
Diarrhea	10 (20.4)	1 (2.0)	11 (17.5)	1 (1.6)	7 (13.0)	1 (1.9)
Proteinuria	16 (32.7)	0	17 (27.0)	2 (3.2)	0	0
Pruritus	2 (4.1)	0	4 (6.3)	0	6 (11.1)	0
Rash	6 (12.2)	2 (4.1)	6 (9.5)	0	8 (14.8)	1(1.9)
Nausea	11 (22.4)	0	15 (23.8)	0	3 (5.6)	0
Vomiting	10 (20.4)	0	14 (22.2)	0	2 (3.7)	0
Abdominal pain	8 (16.3)	0	9 (14.3)	0	1 (1.9)	0
Hypothyroidism	10 (20.4)	0	4 (6.3)	0	5 (9.3)	1 (1.9)
Neutropenia	17 (34.7)	1 (2.0)	12 (19.0)	0	2 (3.7)	0
Thrombocytopenia	18 (36.7)	2 (4.1)	13 (20.6)	0	3 (5.6)	0
Anemia	17 (34.7)	2 (4.1)	18 (28.6)	1 (1.6)	3 (5.6)	0
AST/ALT increase	14 (28.6)	2 (4.1)	15 (23.8)	3 (4.8)	1 (1.9)	0

TRAEs, treatment-related adverse events; ALT, alanine aminotransferase; AST, aspartate aminotransferase.

## Discussion

To the best of our knowledge, this is the first study that evaluated the efficacy and safety of apatinib plus PD-1 mAb as third-line or later treatment for patients with AGC. Compared with the monotherapy of either apatinib or PD-1 mAb, apatinib plus PD-1 mAb treatment yielded longer PFS and OS, and achieved significant higher ORR and DCR. In addition, most of the treatment-related toxicities were mild and tolerable.

Although chemotherapy has been the backbone therapy for AGC for many years, treatment has shifted from chemotherapy to combination therapy including targeted therapy and immunotherapy (17). At present, the approved therapeutic strategies in third line for AGC in China are apatinib and PD-1 mAb monotherapy (10–12). However, apatinib or PD-1 mAb monotherapy has very limited prolongation of survival and cannot meet clinical treatment needs. Additionally, most gastric cancers are not sensitive to PD-1 mAb monotherapy. Therefore, patients with AGC will probably require combination therapy to improve the response rates and duration of immunotherapies.

Substantial preclinical evidence suggests that anti-angiogenic therapy can enhance anti-tumor immunity as it restores function and enhances infiltration of effector T cells, decreases the number of immunosuppressive regulatory T cells (Tregs), tumor- associated macrophages (TAMs) and mast cells, and inhibits the accumulation and the activity of myeloid-derived suppressor cells (MDSCs) (18, 19). Combinatorial approaches investigating PD-1 mAb and angiogenesis inhibitors have been preliminarily validated in several clinical trials. Several phase I/II trials of ramucirumab plus nivolumab or pembrolizumab have shown promising efficacy in patients with AGC (20–22). Also, PD-1 mAb combined with multikinase inhibitors targeting VEGF receptors and other receptor tyrosine kinases, such as regorafenib or lenvatinib, have also shown promising results in patients with AGC (23, 24). In the REGONIVO trial, regorafenib combined with nivolumab gained an ORR of 44% as third-line treatment for patients with AGC, and a median PFS of 5.6 months, and a median OS of 12.3 months (23). Also, a phase 2 trial (EPOC1706) of lenvatinib plus pembrolizumab showed that ORR was 69% and median PFS was 7.1 months for AGC patients in first- or second line treatment (24). The main limitations of these previous studies were not randomization, limited sample size, and high selection of patients with very good ECOG PS.

In the present study, we compared the efficacy and tolerability of apatinib plus PD-1 mAb and apatinib or PD-1 mAb monotherapy. Most of the included patients (75.9%) had ECOG PS of 0 or 1. Almost all of the included patients (93.4%) had received two previous lines of treatment. The median PFS and OS in the overall population were 3.3 months and 7.8 months, respectively, which were longer than the PFS and OS in patients who received chemotherapy in other studies (25–27). Both PFS and OS were significant longer with apatinib plus PD-1 mAb than with apatinib or PD-1 mAb monotherapy. In addition, our study showed better outcomes in terms of ORR and DCR with apatinib plus PD-1 mAb than with apatinib or PD-1 mAb monotherapy. The PFS, OS and ORR results of apatinib plus PD-1 mAb in this study were comparable to those of regorafenib plus nivolumab in the REGONIVO trial (23). These results suggest that immunotherapy (PD-1 mAb) combined with antiangiogenic agents is more favourable than single agent in third-line or later treatment of AGC.

We also conducted subgroup analyses according to clinical factors and found that both PFS and OS in most subgroups showed a better survival benefit in apatinib plus PD-1 mAb than in apatinib or PD-1 mAb. Further breakdown of the subgroup of patients with metastasis by the site of metastasis, apatinib plus PD-1 mAb showed no PFS benefit for patients who had liver metastasis and no OS benefit for patients who had lymph nodes metastasis compared to the apatinib monotherapy. This phenomenon was also observed in the REGONIVO study (23), which may be related to the systemic immunosuppressive activity of liver metastases (28, 29). When analyzing the PFS and OS benefits of patients with lung metastases, we found that these patients were favorable to apatinib or PD-1 mAb monotherapy than apaitnib plus PD-1 mAb. However, there were too few patients with lung metastases and the confidence interval was too large to draw firm conclusions. In females, apatinib plus PD-1 mAb failed to significantly improve PFS and OS when compared with PD-1 mAb monotherapy.

To further identify the impact factors of PFS and OS, we performed a multivariate analysis. Apatinib plus PD-1 mAb treatment was an independent prognostic factor for PFS and OS compared with apatinib or PD-1 mAb monotherapy. On multivariate analyses for apatinib plus PD-1 mAb versus apatinib monotherapy, ECOG PS of 0 or 1 was a protective factor and presence of liver metastasis was a detrimental factor for PFS and OS. On multivariate analyses for apatinib plus PD-1 mAb versus PD-1 mAb monotherapy, PD-L1 CPS ≥1 was considered as an independent protective factor for PFS and OS, while ECOG PS of 0 or 1 has positive impact on OS.

In terms of safety, most of the treatment-related toxicities were mild and tolerable. No significant differences in adverse events of any grade were observed between apatinib plus PD-1 mAb and apatinib monotherapy groups, and most adverse events were apatinib-related. The most frequent side effects were hypertension, hand–foot syndrome, and proteinuria. On the other hand, compared with the combination of apatinib and PD-1 mAb, PD-1 mAb monotherapy had fewer adverse events. No treatment related deaths were recorded.

The present study has several limitations that should be considered. Firstly, this is a non-randomized retrospective study with an inevitably selection bias. Secondly, the small sample size, the number of metastasis organs, different PD-1 mAbs, and lack of molecular pathological information in some patients may affect the results. Thirdly, the impact of different treatment after disease progression was not estimated in this study, which will inevitably affect OS analysis. Furthermore, retrospective studies may miss some patient details leading to underestimation of adverse effects. Thus, further large-scale prospective studies are required to determine the clinical efficacy and safety of apatinib plus PD-1 mAb. Several phase 2 studies comparing apatinib plus PD-1 mAb with apatinib monotherapy are ongoing (NCT04174339, NCT05095636, NCT05342389).

In conclusion, this retrospective study demonstrated that in overall population apatinib plus PD-1 mAb conferred benefit over apatinib or PD-1 mAb monotherapy in PFS, OS, ORR and DCR in third-line or later treatment of AGC patients. The safety profile of apatinib plus PD-1 mAb was similar to that of apatinib, and most adverse events were mild and tolerable. We look forward to exploring the efficacy of apatinib plus PD-1 mAb in larger cohorts of patients.

## Data availability statement

The original contributions presented in the study are included in the article/**Supplementary Material**. Further inquiries can be directed to the corresponding author.

## Ethics statement

The studies involving human participants were reviewed and approved by Ethics committee of Affiliated Cancer Hospital of Zhengzhou University and second People’s Hospital of Pingdingshan. Written informed consent for participation was not required for this study in accordance with the national legislation and the institutional requirements.

## Author contributions

QC, YM and HL contributed to conception and design. QC, YM, DW, YH, LZ and DM acquired the data. DW, YM, DM, LZ and HL analyzed and interpreted the data. QC LZ and YH wrote, reviewed, and/or revised the manuscript. All authors contributed to the article and approved the submitted version.

## Funding

This work was supported by the Medical Science and Technology Research Projects of Henan Province 201702257 (to YH). It was also supported by Henan Medical Science and Technology Research Project (212102310338), Henan Medical Science and Technology Project Jointly Built by the Ministry and the Province (LHGJ20190673), and Postdoctoral Research Project of Henan Province (202002077).

## Conflict of interest

The authors declare that the research was conducted in the absence of any commercial or financial relationships that could be construed as a potential conflict of interest.

## Publisher’s note

All claims expressed in this article are solely those of the authors and do not necessarily represent those of their affiliated organizations, or those of the publisher, the editors and the reviewers. Any product that may be evaluated in this article, or claim that may be made by its manufacturer, is not guaranteed or endorsed by the publisher.
