# Supplementary material for: Apatinib combined with PD-1 antibody for third-line or later treatment of advanced gastric cancer
Source: Front Oncol. 2022 Oct 28;12:952494. doi: 10.3389/fonc.2022.952494 (PMC9650409; doi:10.3389/fonc.2022.952494)
Supplement: Supplementary file 1 [file DataSheet_1.pdf]

Table S1. Univariate analyses of progression-free survival and overall survival for apatinib plus PD-1 mAb *versus* apatinib monotherapy

	Progression-free survival			Overall survival		
**Variable**	HR	95%CI	*p* value	HR	95%CI	*p* value
**Age**						
≥65 years	0.809	0.543-1.205	0.297	1.067	0.697-1.634	0.767
<65 years	Ref			Ref		
**Gender**						
Male	0.618	0.213-1.213	0.023	0.569	0.113-0.987	0.016
Female	Ref			Ref		
**Primary site**						
Gastric	1.163	0.767-1.765	0.477	1.132	0.722-1.755	0.590
Gastroesophageal	Ref			Ref		
**Histology**						
Diffuse	1.128	0.753-1.688	0.56	1.397	0.890-2.190	0.146
Intestinal	Ref			Ref		
**ECOG**						
0-1	0.424	0.135-0.976	0.001	0.349	0.196-0.853	0.001
2	Ref			Ref		
**Previous gastrectomy**						
Yes	1.058	0.706-1.586	0.785	1.049	0.675-1.632	0.831
No	Ref			Ref		
**Previous lines of treatment**						
2	0.845	0.343-2.079	0.713	0.986	0.361-2.697	0.978
>2	Ref			Ref		
**Site of metastasis**						
**Lymph node**	1.230	0.764--	0.394	0.952	0.581-1.560	0.846
No	Ref			Ref		
**Liver**	1.981	1.336-2.939	0.001	2.098	1.373-3.206	0.001
No	Ref			Ref		
**Peritoneum**	1.702	1.408-2.617	0.015	0.769	0.485-1.219	0.264
No	Ref			Ref		
**Lung**	1.225	0..730-2.053	0.442	1.184	0.677-3.070	0.576
No	Ref			Ref		
**Her-2 Status**						
Positive	0.902	0.533-1.526	0.701	0.617	0.346-1.101	0.617
Negative	Ref			Ref		
**Treatment**						
Apatinib plus PD-1 mAb	0.569	0.379-0.851	0.006	0.593	0.386-0.912	0.017
Apatinib	Ref			Ref		

HR, hazard ratio; CI, confidence interval; ECOG PS, eastern cooperative oncology group performance status; Ref, reference

Table S2. Univariate analyses of progression-free survival and overall survival for apatinib plus PD-1 mAb *versus* PD-1 mAb monotherapy

	Progression-free survival			Overall survival		
**Variable**	HR	95%CI	*p* value	HR	95%CI	*p* value
**Age**						
⩾65 years	0.880	0.573-1.350	0.557	1.116	0.719-1.733	0.624
<65 years	Ref			Ref		
**Gender**						
Male	0.764	0.490-1.191	0.235	0.687	0.433-1.091	0.112
Female	Ref			Ref		
**Primary site**						
Gastric	1.287	0.814-2.036	0.280	0.911	0.572-1.449	0.693
Gastroesophageal	Ref			Ref		
**Histology**						
Diffuse	0.965	0.633-1.469	0.867	0.964	0.618-1.504	0.872
Intestinal	Ref			Ref		
**ECOG**						
0-1	0.665	0.403-1.095	0.109	0.644	0.384-1.078	0.094
2	Ref			Ref		
**Previous gastrectomy**						
Yes	0.768	0.497-1.186	0.233	0.675	0.422-1.078	0.100
No	Ref			Ref		
**Previous lines of treatment**						
2	0.853	0.412-1.768	0.670	0.621	0.309-1.248	0.181
>2	Ref			Ref		
**Site of metastasis**						
**Lymph node**	0.966	0.594-1.571	0.889	0.724	0.440-1.160	0.204
No	Ref			Ref		
**Liver**	1.414	0.923-2.169	0.112	1.148	0.736-1.789	0.544
No	Ref			Ref		
**Peritoneum**	1.243	0.786-1.965	0.353	1.187	0.733-1.922	0.486
No	Ref			Ref		
**Lung**	1.476	0..857-2.544	0.161	1.690	0.973-2.933	0.062
No	Ref			Ref		
**Her-2 Status**						
Positive	1.081	0.649-1.801	0.765	1.182	0.710-1.967	0.520
Negative	Ref			Ref		
**PD-L1 CPS**						
⩾1	0.360	0.229-0.5666	0.001	0.243	0.143-0.412	0.001
<1	Ref			Ref		
**Treatment**						
Apatinib plus PD-1 mAb	0.621	0.405-0.951	0.028	0.554	0.352-0.871	0.011
PD-1 mAb	Ref			Ref		

HR, hazard ratio; CI, confidence interval; ECOG PS, eastern cooperative oncology group performance status; Ref, reference; CPS, combined positive score
